# VDR gene variants FokI and ApaI: Factors associated with susceptibility to multiple sclerosis

**DOI:** 10.1371/journal.pone.0332473

**Published:** 2025-09-17

**Authors:** Laith AL-Eitan, Salma Darabseh

**Affiliations:** 1 Department of Applied Biological Sciences, Jordan University of Science and Technology, Irbid, Jordan; 2 Department of Biotechnology and Genetic Engineering, Jordan University of Science and Technology, Irbid, Jordan; Universitas Muhammadiyah Aceh, INDONESIA

## Abstract

Multiple Sclerosis (MS) is a commonly observed autoimmune inflammatory condition that affects the central nervous system (CNS). Vitamin D functions as a steroid hormone by interacting with its nuclear receptor, the vitamin D receptor (VDR), to regulate critical biological processes. The polymorphisms of the *VDR* gene have not yet been investigated in the cohort of MS patients in Jordan. We aimed to examine the genetic associations between polymorphisms in the *VDR* gene (specifically, *TaqI, BsmI, ApaI, and FokI*) and susceptibility to Multiple Sclerosis (MS). Additionally, we aimed to investigate the relationship between vitamin D status and *VDR* gene polymorphisms in relation to the onset of MS in Jordanian individuals. The study cohort included 218 individuals diagnosed with Multiple Sclerosis (MS) and 200 healthy controls. The Sequenom MassARRAY system was used for genotyping all single-nucleotide polymorphisms (SNPs). The findings reveal a significant correlation, indicating an increased risk of multiple sclerosis associated with *FokI* (*P* = 0.03) and *ApaI* (*P *= 0.04), contrasting with the findings for *BsmI* and *TaqI*. Only the *FokI* SNP has been significantly linked (P = *0.03*) to a clinical phenotype of multiple sclerosis: vitamin D deficiency. While the cross-sectional nature of the study limits causal interpretations, the results highlight the potential role of the Vitamin D Receptor gene in MS susceptibility. Further studies on gene-environment interactions should be conducted in a distinct population of Arab descent to strengthen and validate the genetic link between VDR and MS susceptibility.

## Introduction

Multiple sclerosis (MS), an idiopathic inflammatory disease of the central nervous system, is pathologically characterized by demyelination and subsequent axonal degeneration [1]. It is widely recognized as an autoimmune condition in which the immune system targets the myelin sheath, leading to chronic inflammation and a distinct element of neurodegeneration [2]. Multiple sclerosis (MS) typically affects individuals between the ages of 20 and 50, with an average age of onset around 30 years. However, it can also occur in early childhood or later in life, even after the age of 60. MS is significantly more common in females, with a prevalence approximately three times higher in women than in men [3]. According to the latest data from the Atlas of MS, as of 2020, approximately 2.8 million people worldwide live with MS, equating to a prevalence of about 35.9 per 100,000 individuals [4]. The prevalence of MS exhibits a geographical pattern characterized by a latitudinal gradient and an irregular distribution. According to the most recent epidemiological study on individuals with multiple sclerosis in Jordan, the country is classified as having a medium-to-high risk for MS, characterized by a prevalence rate of 39 cases per 100,000 individuals [5]. Genetics and environmental factors are the major known causes of MS [6]. Environmental factors of significance may include exposure to the Epstein–Barr virus (EBV), symptoms of infectious mononucleosis, limited sunlight exposure, vitamin D deficiencies, and cigarette smoking habits [7]. Recent epidemiological data substantiate a connection between diminished levels of vitamin D and heightened risk of chronic diseases, encompassing cancer, autoimmune conditions like systemic lupus erythematosus, and type 1 diabetes, along with cardiovascular disease, including multiple sclerosis [8,9]. Supporting its immunomodulatory role, a study in Indonesian newborns revealed that vitamin D levels influence inflammatory cytokines such as IL-6 and IL-10, suggesting its involvement in the balance between pro- and anti-inflammatory responses early in life. However, linear associations were not consistently significant [10].

Vitamin D, a fat-soluble pro-hormone, can be acquired from dietary sources or synthesized in the skin. Upon synthesis, it undergoes metabolic conversion in the liver, forming 25-hydroxyvitamin D [25(OH)D]. This metabolite acts as the primary circulating form of vitamin D and functions as a reliable marker for assessing an individual’s vitamin D status [11]. Within the kidney, 25-hydroxyvitamin D undergoes enzymatic conversion into its biologically active form, 1,25-dihydroxyvitamin D. This active metabolite assumes a pivotal role in the regulation of a diverse range of biological processes, including but not limited to calcium homeostasis, bone formation, cell growth, proliferation, apoptosis, and maintenance of immune system balance [12]. Notably, apart from the kidney, various cell types, including immune cells, can synthesize 1,25(OH)2D, the primary ligand responsible for activating vitamin D and binding to the nuclear vitamin D receptor (VDR). This interaction has significant implications for modulating the immune system [13]. The Vitamin D Receptor is a member of the steroid/thyroid nuclear receptor superfamily and is expressed in a wide range of cell types, including those of the immune system. It regulates gene expression by binding to DNA via zinc finger domains and interacting with various co-regulatory proteins [11,13]. The human VDR gene is located on chromosome 12 (12q13.11) and comprises 12 exons and 11 introns.

Specific variations within the VDR gene have been associated with alterations in the functionality and metabolism of vitamin D [14]. While the VDR gene exhibits over 30 identified polymorphisms, these genetic variations could form a genetic basis for susceptibility to Multiple Sclerosis (MS), providing valuable insights into the relationship between Vitamin D and MS [15]. The most extensively studied polymorphisms related to MS are commonly denoted by the names of the restriction enzymes used for genotyping. The *ApaI* (rs7975232), *BsmI* (rs1544410), and *TaqI* (rs731236) polymorphisms are situated near the 3’ end of the *VDR* gene, whereas the *FokI* (rs2228570) polymorphism is positioned near the 5’ end of the gene [16]. Numerous investigations have explored the relationship between polymorphisms within the *VDR* gene and susceptibility to Multiple Sclerosis [17,18]. However, these findings remained inconclusive, emphasizing the necessity for a comprehensive study that examines the genetic and environmental influences of vitamin D on MS. To address this issue, we conducted a case-control study to clarify the impact of four specific VDR genetic polymorphisms in individuals from Jordan affected by multiple sclerosis (MS). Additionally, we evaluated the concentrations of 25-hydroxyvitamin D (25(OH)D) in the Jordanian population to determine the potential connection between VDR polymorphisms and the clinical characteristics of the disease.

## Materials and methods

### Subjects

A total of 418 subjects were recruited for this study, including 218 individuals clinically diagnosed with multiple sclerosis (MS) according to the McDonald criteria (2005) [19]. The 2005 McDonald criteria were employed in this study to maintain consistency with earlier clinical evaluations and patient records, as a substantial proportion of participants were diagnosed and enrolled during a period when these criteria were standard in local clinical practice. This approach also facilitated comparison with previously published regional studies that used the same criteria. Nonetheless, we acknowledge this as a limitation, given that the 2010 and 2017 revisions of the McDonald criteria introduced improved diagnostic sensitivity and reflect more recent advances in the understanding and diagnosis of MS. In addition to the MS group, 200 age-matched control participants without neurological or systemic disorders were included. DNA samples from patients and control individuals were obtained through Prof. Al-Eitan, who is conducting research on the genetic association of MS in Jordanian patients. The Institutional Review Board (IRB) of Jordan University of Science and Technology granted ethical approval under the ethical code 12/108/2017, dated August 29, 2017. Patient recruitment was conducted over one year, from January 21, 2018, to January 21, 2019, across multiple healthcare facilities, including Princess Basma Hospital, King Abdullah University Hospital, Jordanian Royal Medical Services, and Al-Basheer Hospital. The current study has also received another ethical approval (Ethical Approval #: 28/121/2019, dated February 28, 2019) from the IRB committee at Jordan University of Science and Technology to conduct this continuation study on the genetic association of multiple sclerosis (MS) in Jordanian patients, covering the period from March 31, 2019, to February 12, 2020.

The exclusion criteria for the case group included individuals with an unverified diagnosis of Multiple Sclerosis (MS), those with first or second-degree relatives participating in the study, non-Jordanian participants, those with an aversion to blood or needles, individuals who did not provide informed written consent, and those with restricted or absent clinical data. In contrast, the inclusion criteria for the control group required participants to be healthy Jordanians with no reported history of neurological, autoimmune, or other chronic diseases. Additionally, individuals with first- or second-degree relatives diagnosed with MS or those who had previously undergone vitamin D-level testing were excluded from the control group.

### Vitamin D measurement

Serum 25-OH vitamin D levels were measured in both cases and the healthy control group using the MAGLUMI 25-OH vitamin D (CLIA) kit. The MAGLUMI 25-OH vitamin D (CLIA) kit is specifically engineered for in vitro chemiluminescence immunoassays, offering a precise method for quantifying 25-OH vitamin D levels in human serum. The analysis used the MAGLUMI series fully automated chemiluminescence immunoassay analyzer, notably the Maglumi 800 model. This laboratory analysis was conducted at the Biotechnology and Genetic Engineering Laboratory at JUST, located in Irbid, Jordan.

### The candidate gene and the SNPs

Many genes have been proposed as candidate genes for susceptibility to MS. To identify Single Nucleotide Polymorphisms (SNPs) that could potentially be linked to an elevated MS risk within the Jordanian population, one candidate gene was chosen for investigation in this study, primarily based on considerations that suggest its potential involvement in certain aspects of multiple sclerosis and therefore vitamin D status. After the *VDR* gene was chosen, four SNPs were identified within this gene. SNPs rs2228570 [20], rs1544410 [21], rs731236 [20], and rs7975232 [22] have been thoroughly studied for their association with MS susceptibility and development in different populations. Illustrative gel electrophoresis results of PCR-restriction fragment length polymorphism products for all SNPs were provided in S1–S4 Figs.

### The extraction of DNA and the genotyping of single-nucleotide polymorphisms (SNPs)

Genomic DNA extraction was performed on peripheral blood samples collected in EDTA tubes from both study groups using the Gentra Puregene Blood Kit by Qiagen, Germany, according to the manufacturer’s protocols. The evaluation of both the quality and quantity of the isolated genomic material was conducted using two methodologies: the Nano-Drop (version ND-1000, manufactured by Bio Drop in the UK) and gel electrophoresis. The DNA samples were forwarded to the Australian Genome Research Facility (AGRF) at the Melbourne Node in Melbourne, Australia. The samples were sent to genotype the VDR single-nucleotide polymorphisms (SNPs) using the Sequenom MassARRAY® system (iPLEX GOLD), a technology developed by Sequenom based in San Diego, CA, USA.

The analytical process involved identifying polymorphisms, which began with amplifying the specific gene region using the polymerase chain reaction. Subsequently, restriction fragment length polymorphism (RFLP) analysis was employed. Then, 10 µL of each digested PCR product and 5 µL of 50 bp and 100 bp ladders were separated using a 3% agarose gel. A total of 5 µL of 10 mg/mL ethidium bromide was added to the gel for staining the bands. Table 1 presents the PCR product sizes and the corresponding restriction fragment sizes for all analyzed SNPs. The optimized thermal cycling conditions, including the annealing temperatures specific to each SNP, are also indicated following the optimization of the polymerase chain reaction thermal profile.

**Table 1:** PCR product size of DNA and restriction fragment sizes for all SNPs.

VDR gene polymorphisms	Alleles	PCR program (35 cycles)	PCR Product Size	Restriction Endonuclease Incubation temperature	Fragments Size (bp)
**Fok-I**	**T/C**	**94** ^**o**^ **C for 1 min, 60** ^**o**^ **C 1 min 72** ^**o**^ **C for 1 min**	**265 bp**	***FokI***	**TT**:196–69 **TC**:265–196–69 **CC**:265
**Bsm-I**	**C/T**	**95** ^**o**^ **C for 1 min, 60** ^**o**^ **C 1 min 72** ^**o**^ **C for 1 min**	**273 bp**	***BsmI***	**CC**: 273 **CT**: 273–189–75 **TT**: 189–75
**Taq-I**	**G/A**	**93** ^**o**^ **C for 45s, 56** ^**o**^ **C 30s, 72** ^**o**^ **C for 45s**	**750 bp**	***TaqI***	**GG**:570–60 **GA**:750–570–60 **GG**:750
**Apa**	**C/A**	**93** ^**o**^ **C for 45s, 56** ^**o**^ **C 30s, 72** ^**o**^ **C for 45s**	**750 bp**	***ApaI***	**AA**:570–60 **CA**:750–570–60 **CC**:750

### Statistical analysis

The statistical analysis was conducted using SPSS version 25 (IBM SPSS Statistics, Chicago, IL). Genotype and allele frequencies were determined through the Chi-square (χ2) test, and descriptive statistics, presented as mean values with standard deviations, were analyzed using analysis of variance (ANOVA). An assessment of deviation from Hardy-Weinberg Equilibrium was conducted to examine the distribution of genotypes and alleles within both the patient and healthy control groups. This analysis was performed using the SNPstats tool, available at https://www.snpstats.net/start.htm. Furthermore, odds ratios (OR) and their corresponding 95% confidence intervals (CI) were computed. Statistical significance was set at a *P value* *<* *0.05*. To improve the validity of the genetic association findings, multiple testing was accounted for using the Bonferroni correction, wherein the significance threshold (α = 0.05) was adjusted based on the number of independent comparisons.

### Machine learning analysis

The association between VDR genotypes and vitamin D3 levels in MS patients was investigated using a linear regression model implemented through the machine learning tool Weka (version 3.8).

## Results

### Descriptive analysis

A total of 418 participants were enrolled in this study, comprising 218 individuals diagnosed with multiple sclerosis (MS) and 200 age-matched healthy controls. The MS cohort consisted of 147 females and 71 males, with a mean age of 35.70 ± 10.20 years. The healthy control group consisted of 138 females and 62 males, with a mean age of 27.08 ± 6.19 years. A statistically significant age difference was observed between the two groups (*P* *<* *0.05*). Regarding MS disease characteristics, the cohort predominantly consisted of individuals with relapsing-remitting multiple sclerosis (RRMS), the most common form of MS, accounting for approximately 87% of cases. The mean disease duration was 8.5 ± 5.3 years. A significant difference in vitamin D deficiency status was observed between patients and controls (P < 0.05). Detailed demographic and clinical data for both groups are summarized in Table 2.

**Table 2:** Description of demographic and clinical characteristics of MS patients and healthy controls.

Characteristic	Mean ± SD with %	
	Patient	Control
Number	218	200
Male Female	71 (32.6%) 147 (67.4%%)	62 (31.0%) 138 (69.0%)
Age (years)	35.70 ± 10.20	27.08 ± 6.19
BMI (kg/m2)	24.7 **± **5.3	24.8 **± **4.1
Clinical data		
Vitamin D Deficiency		
Yes	93.4%	37.24%
No	6.6%	62.76%

### Hardy–Weinberg Equilibrium (HWE) analysis

After subjecting the assessed polymorphisms to the HWE test within both the case and control sample cohorts, it was observed that all the examined single-nucleotide polymorphisms (SNPs) adhered to the HWE criteria in both the case and control groups. As a result, these SNPs were incorporated into the study. Table 3 provides detailed information on the minor alleles of the investigated SNPs and their respective frequencies in both the case and control groups. The results indicated no statistically significant association with the risk of Multiple Sclerosis for any of the VDR gene single-nucleotide polymorphisms (SNPs) within the case and control groups.

**Table 3:** VDR SNPs, their minor allele frequencies, and HWE P-values in cases and controls.

Gene	SNP _ID	Cases (n = 218)			Controls (n = 200)		
		MA^a^	MAF^b^	HWE^c^*p-value*	MA^a^	MAF^b^	HWE^c^*p-value*
***VDR***	rs2228570	T	31%	**0.019** ^**Δ**^	T	25%	1
	rs1544410	T	40%	0.32	T	44%	0.2
	rs731236	G	37%	0.25	G	42%	0.19
	rs7975232	C	41%	0.48	C	34%	0.87

^a^MA: Minor allele.

^b^MAF: Minor allele frequency.

^c^HWE: Hardy-Weinberg equilibrium.

^**Δ**^Significant P values are indicated by a triangle (^**Δ**^ P < 0.05).

### Exploring the relationship between SNPs in candidate genes and multiple sclerosis

The genotype frequencies of the *FokI* SNP (rs2228570(T/C)), located proximal to the translation initiation codon within exon number 2 of the Vitamin D Receptor gene on chromosome 12, demonstrated an association with susceptibility to Multiple Sclerosis (MS). The genotype frequency in the MS cases group showed a significant difference from that of the control group, revealing an overall estimated odds ratio of 2.48 (χ2 (2, N = 418) = 6.43, *P-value* = 0.04). Furthermore, notable distinctions were evident in the allele frequencies between MS cases and controls (*P =* 0.03 for allele frequency). Specifically, the T allele of rs2228570 exhibited a higher prevalence in cases (31%) compared to controls (25%). These findings indicated that the T allele was the risk allele for MS in this study, as detailed in Table 4. The heterozygous TC genotype and homozygous CC genotype were less common among MS cases (36% and 51%, respectively) than in the control group (38% and 56%, respectively). In contrast, the homozygous TT genotype had a higher prevalence among MS cases (13%) than a lower frequency of 6% observed among the control group.

**Table 4:** Association of genes SNPs with multiple sclerosis susceptibility.

Gene	SNP_ID	Allele/ Genotype	Cases (218)	Controls (200)	Cases vs. Controls	
					Chi-squared	*P* -value
***VDR***	rs2228570	C	299 (69%)	301 (75%)	4.58	0.03 ^Δ^
		T	137 (31%)	99 (25%)		
		CC	110 (50%)	113 (56%)	6.43	0.04 ^Δ^
		TC	79 (36%)	75 (38%)		
		TT	29 (13%)	12 (6%)		
	rs1544410	C	263 (60%)	225 (56%)	1.42	0.23
		T	173 (40%)	175 (44%)		
		CC	83 (38%)	68 (34%)	1.37	0.50
		CT	97 (44%)	89 (44%)		
		TT	38 (17%)	43 (22%)		
	rs731236	A	274 (63%)	232 (58%)	2.05	0.15
		G	162 (37 %)	168 (42%)		
		AA	90 (41%)	72 (36%)	1.91	0.38
		AG	94 (43%)	88 (44%)		
		GG	34 (16%)	40 (20%)		
	rs7975232	A	258 (59%)	264 (66%)	4.14	0.04 ^Δ^
		C	178 (41%)	136 (34%)		
		AA	79 (36%)	86 (43%)	4.60	0.10
		CA	100 (46 %)	92 (46%)		
		CC	39 (18%)	22 (11%)		

Δ Significant P values are indicated by a triangle (^**Δ**^ P < 0.05).

P-values < 0.0062 (0.05/8, after applying Bonferroni correction for multiple comparisons) were considered statistically significant and are indicated in bold.

Moreover, the *ApaI* SNP (rs7975232 C/A), situated within an intron region of the VDR gene on chromosome 12, exhibited a significant association with MS susceptibility in terms of allele frequencies, indicating an overall estimated odds ratio of 1.93 (χ2 (2, N = 418) = 4.14, *P-value* = 0.04). In this case, the rs7975232 – C allele was more prevalent among cases (41%) than controls (34%). These results indicated that the C allele was the risk allele for MS in this study, as shown in Table 4. Nevertheless, there were no significant differences in the genotype frequencies between MS cases and controls (*P* = 0.1 for genotype frequency). The homozygous CC genotype was more prevalent among MS cases (18%) compared to controls (11%). On the contrary, the allele and genotype frequencies of the *TaqI* SNP (rs731236 G/A) and *BsmI* SNP (rs1544410 C/T), situated in the synonymous and intron variant regions of the *VDR* gene on chromosome 12, did not show significant differences between MS patients and healthy controls, as outlined in Table 4. All SNPs failed to reach statistical significance after applying correction for multiple comparisons.

### Genetic association of *FokI* and *ApaI* SNPs with multiple sclerosis utilizing diverse genetic models

In the context of the *FokI* SNP, no genetic correlations were detected when employing the dominant genetic model, which contrasts the prevalent homozygous CC genotype against the heterozygous TC and infrequent homozygous TT genotypes. This inference is drawn from the Chi-square statistical analysis, where the obtained chi-square values indicate that the P-value is greater than 0.05. However, when applying the recessive genetic model, a significant association was observed between the prevalent homozygous CC and the heterozygous TC genotypes compared to the rare homozygous TT genotype, as indicated by the Chi-square test results. Similar to the *FokI* SNP, the dominant genetic model of the *ApaI* SNP did not reveal any genetic associations. Specifically, no significant associations were identified when comparing the common homozygous AA genotype with the heterozygous CA genotype and the rare homozygous CC genotype, as indicated by the Chi-square results, which showed chi-square values with p-values greater than 0.05. However, in the recessive genetic model, a marginal but statistically discernible association was observed when comparing the common homozygous AA genotype and the heterozygous CA genotype to the rare homozygous CC genotype, as demonstrated by the Chi-square results. All SNPs failed to reach statistical significance after applying correction for multiple comparisons.

Genetic association analysis of genotypes for the four SNPs in MS cases and controls was conducted using a range of genetic statistical methodologies, including both dominant and recessive genetic models of the four Nucleotide Polymorphisms of interest. The statistical analysis results for the genetic association of each of the examined SNPs are presented in Table 5. This analysis involved comparing the heterozygous genotype to the common homozygous genotype, contrasting it with the rare homozygous genotype, and assessing the association between the common homozygous and rare homozygous genotypes.

**Table 5:** Genetic models and distributions of SNPs within the VDR gene in 218 cases and 200 controls.

Gene	SNP ID	Model	Cases (%)	Controls (%)	OR (95% CI)	p-Value *
***VDR***	**rs2228570**	C/C**/** (T/C+T/T) (C/C+T/C)**/** T/T	50.5%/49.5% 86.7%/13.3%	56.5%/43.5% 94%/6%	1.28 (0.87-1.88) 2.40 (1.19-4.85)	0.22 0.01 ^Δ^
	**rs1544410**	C/C**/** (C/T+T/T) (C/C+C/T)**/** T/T	82.6%/17.4%	34%/66% 78.5%/21.5%	0.84 (0.56-1.25) 0.77 (0.47-1.25)	0.39 0.29
	**rs731236**	A/A/ (G/A+G/G) (A/A+G/A)/ G/G	41.3%/58.7% 84.4%/15.6%	36%/64% 80%/20%	0.80 (0.54-1.19) 0.74 (0.45-1.22)	0.27 0.24
	**rs7975232**	A/A/ (C/A+C/C) (A/A+C/A)/ C/C	36.2%/63.8% 82.1%/17.9%	43%/57% 89%/11%	1.33 (0.90-1.97) 1.76 (1.00-3.09)	0.16 0.04 ^Δ^

^**Δ**^Significant P values are indicated by a triangle (^**Δ**^ P < 0.05) .

P-values < 0.0062 (0.05/8, after applying Bonferroni correction for multiple comparisons) were considered statistically significant and are indicated in bold.

### Analysis of the haplotypes within the VDR gene

Regarding the haplotype frequencies within the VDR gene, the analysis of quaternary allelic haplotypes, as presented in Table 6, revealed a significant association (CCGA, P < 0.0001) and (CTAA, P = 0.0001) for *FokI, BsmI, TaqI,* and *ApaI* SNPs with multiple sclerosis patients and controls. Following multiple testing correction, the CCGA and CTAA haplotypes retained statistical significance.

**Table 6:** Frequencies of the VDR gene haplotypes among the 218 MS patients and 200 healthy controls.

Gene	Haplotypes^a^	Frequency (cases/control)	Odds Ratio (95% CI)	*p*-value
***VDR***	CCAC	(0.29)/ (0.13)	1	---
	CTGA	(0.24)/ (0.10)	0.76 (0.40 - 1.45)	0.41
	CCAA	(0.13)/ (0.09)	0.57 (0.28 - 1.18)	0.13
	TTGA	(0.13)/ (0.05)	1.74 (0.62 - 4.84)	0.29
	CCGA	(0.002)/ (0.21)	0.01 (0.00 - 0.06)	**<0.0001** ^Δ^
	TCAC	(0.12)/ (0.05)	0.95 (0.36 - 2.50)	0.92
	CTAA	(0.03)/ (0.11)	0.14 (0.06 - 0.38)	**0.0001** ^Δ^
	TCAA	(0.06)/ (0.03)	0.54 (0.21 - 1.41)	0.21

^a^Loci of tag SNPs are written 3′ to 5′and include the following SNPs: rs2228570, rs1544410, rs731236, and rs7975232 in VDR, gene.

Δ Significant P values are indicated by a triangle (^**Δ**^ P-value).

P-values < 0.0062 (0.05/8, after applying Bonferroni correction for multiple comparisons) were considered statistically significant and are indicated in bold.

### Genotype versus phenotype correlation among MS patients

The summary presented in Table 7 provides insights into the associations between Vitamin D Receptor SNPs and various clinical characteristics in Jordanian patients with MS. Notably, only the *FokI* SNP (rs2228570) exhibited a statistically significant association with vitamin D deficiency, as indicated by a *P*-value of 0.03. In contrast, none of the other VDR single-nucleotide polymorphisms exhibited significant associations with the clinical characteristics of MS, as shown in Table 7. No statistically significant SNPs were identified following adjustment for multiple comparisons.

**Table 7:** Association between different VDR SNP genotypes and multiple sclerosis (MS) clinical characteristics.

Clinical characteristics	VDR			
	rs2228570 CC/TC/TT	rs1544410 CC/CT/TT	rs731236 AA/GA/GG	rs7975232 AA/CA/CC
**Body mass index** ^**^	0.503	0.151	0.147	0.184
**Vit. D deficiency** ^**^	0.030 ^**Δ**^	0.999	0.555	0.839
**Patient age** ^**^	0.757	0.864	0.903	0.928
**Gender** ^*^	0.527	0.512	0.653	0.365
**Height** ^**^	0.409	0.557	0.540	0.395
**Weight** ^**^	0.155	0.060	0.060	0.070

* Pearson’s Chi-squared test was used to determine genotype-phenotype association.

** Analysis of variance (ANOVA) test was used to determine genotype-phenotype association.

^**Δ**^Significant P values are indicated by a triangle (^**Δ**^ P < 0.05) .

P-values < 0.0021 (0.05/24, after applying Bonferroni correction for multiple comparisons) were considered statistically significant and are indicated in bold.

The Weka machine learning using classifier function (linear regression) was used to test genotype vs. vitamin D deficiency phenotype for the four SNPs within the *VDR* gene among MS patients. The correlation coefficient obtained from the Linear regression model output reveals that only the *FokI* SNP demonstrates a notable association with vitamin D deficiency in individuals with Multiple Sclerosis. The following linear equation represents the relationship: vitaminDdeficiency=6.34+1.82*(rs2228570). The classification of Vitamin D levels with VDR SNP genotypes is shown in S5 Fig.

## Discussion

This study aimed to investigate the association between vitamin D receptor (VDR) gene polymorphisms—specifically *Fok-I* (rs2228570) and *Apa-I* (rs7975232)—and the susceptibility to multiple sclerosis (MS) in a Jordanian cohort. The research aimed to investigate whether these genetic variants contribute to MS risk in this population, considering the established role of vitamin D in immune modulation and the potential impact of VDR polymorphisms on vitamin D function. In our current investigation, a statistically significant difference in the frequency of the *Fok-I* polymorphism was observed between the control group and the patients, yielding a *p-*value of 0.04. This distinction was further substantiated by significant differences in allele frequencies, with a *p*-value of 0.03. Moreover, a notable difference in allele frequencies for the *Apa-I* SNP was observed between the control group and the patients, yielding a *p*-value of 0.04. These results are consistent with the findings of a case-control study by researchers in Turkish and Portuguese MS patients [22,23]. The SNP *rs2228570*, considered a potential causal variant, has been demonstrated to exert a functional impact on gene expression. The *Fok-I* polymorphism in exon two can alter the VDR protein’s structure and influence its transcriptional activity [24–26]. Significantly, the association was detected in the standard homozygous CC and heterozygous CT models when compared to the rare homozygous TT, yielding a *P*-value of 0.01 for rs2228570. Moreover, a significant association was revealed by comparing genotypic frequencies between the standard homozygous AA, heterozygous CA, and rare homozygous AA, resulting in a *P*-value of 0.04 for rs7975232. These findings provide supportive evidence suggesting that the *FokI* and *ApaI* SNPs within the VDR gene may contribute to the development of multiple sclerosis in the Jordanian population. Although the precise biological mechanisms underlying VDR gene involvement in MS remain fully elucidated, our analysis of the *TaqI* polymorphism (rs731236) revealed no significant differences in either genotypic or allelic frequencies between MS patients and controls. These findings are consistent with a study conducted among Caucasian MS patients in Greece [27]. It is worth noting that the *TaqI* polymorphism, located in the intronic region between exons 8 and 9 of the VDR gene, does not result in structural changes to the VDR protein [28], which may explain the lack of functional impact observed. Similarly, the *BsmI* (C/T) genotyping results did not demonstrate a significant association with MS susceptibility in our cohort, aligning with the conclusions of a prior meta-analysis [29]. Nonetheless, other studies have reported a significant correlation between *BsmI* polymorphism and MS risk [30], suggesting that discrepancies in findings may be attributed to ethnic and geographical variability among study populations. Significantly, our investigation revealed a notable association between vitamin D deficiency and the VDR *Fok-I* variant, particularly in relation to the T (f) allele, suggesting a potential genotype-phenotype interaction in the context of multiple sclerosis susceptibility (*P* *=* *0.03***).** This finding is consistent with a study by researchers [31], which also observed an increased risk of MS among individuals carrying the *Fok-I* polymorphism combined with low vitamin D intake. Previous research has suggested that the *Fok-I* polymorphism may affect immune regulation and influence circulating levels of both 25(OH)D and 1,25(OH)₂D [32]. At the molecular level, the T (f) allele introduces a *Fok-I* restriction enzyme site, leading to a VDR protein that is three amino acids longer (427 amino acids) than that encoded by the C (F) allele (424 amino acids). The shorter, wild-type protein (F allele) has been associated with greater transcriptional activity and more efficient protein-protein interactions [33]. Despite extensive exploration of VDR gene polymorphisms in MS, the results across studies remain inconsistent. This variability may be attributed to several factors, including ethnic and geographical diversity, potential gene-environment interactions, sample size limitations, and clinical heterogeneity across cohorts.

This study offers several notable strengths. First, it is among the few investigations to examine the association between VDR gene polymorphisms (*FokI* and *ApaI*) and multiple sclerosis susceptibility in a Middle Eastern population, addressing an underrepresented demographic in genetic MS research. Second, including both genotypic data and serum 25(OH)D levels provides a more comprehensive understanding of the gene-environment interaction, particularly the interplay between genetic predisposition and vitamin D deficiency. Additionally, the relatively balanced sample size (218 patients vs. 200 controls) adds value regarding case-control comparability.

However, several limitations should be acknowledged. The cross-sectional design restricts the ability to infer causal relationships between VDR polymorphisms, vitamin D status, and MS development. One notable limitation is the method initially used for sample size estimation, which was based on disease prevalence using the “Frequency in a Population” option in OpenEpi. While suitable for prevalence studies, this approach may not provide sufficient statistical power to detect genetic associations in a case-control setting. Although our study included more MS cases than the minimum calculated, the sample size may still be inadequate for identifying small to moderate genetic effects, particularly for less common haplotypes. This limited power may have contributed to non-significant findings in certain SNP-specific combinations.

Additionally, the sample size may limit the detection of subtle subgroup interactions. Clinical heterogeneity (e.g., MS subtype and disease duration) was not fully captured, and the functional validation of the identified variants was beyond the scope of the study. Lastly, given the potential population-specific nature of our findings, caution is warranted when generalizing the results to other ethnic groups, and replication in larger, multiethnic cohorts is recommended.

In summary, while this research contributes to our understanding of MS susceptibility in a Middle Eastern context, future studies with larger sample sizes, functional assays, and diverse populations are essential to validate these findings and clarify the precise role of VDR variants in MS pathogenesis.

## Conclusion

In conclusion, this study reinforces the gene-environment interactions model of MS. The Vitamin D Receptor gene plays a critical role in susceptibility to MS, as evidenced by statistically significant *p*-values (*P *< 0.05). Notably, the SNPs (*Fok-I, p-value =* 0.03) and (*Apa-I*, *p-value =* 0.04) within the Vitamin D Receptor gene are correlated with an elevated likelihood of developing multiple sclerosis. Finally, the significance and novelty of this study stem from the fact that genetic diversity within Arab populations is often underrepresented in global research. Our study addresses this gap by examining VDR polymorphisms in relation to multiple sclerosis susceptibility in a region where such data remain scarce. Understanding genetic variations in distinct ethnic groups is essential for advancing personalized medicine and developing targeted therapeutic strategies. Our findings lay the groundwork for further exploration of genetic factors influencing disease predisposition among Arab populations, with potential implications for future clinical and epidemiological research. Additional studies investigating gene-environment and gene-gene interactions, mainly focusing on vitamin D metabolizing genes, are warranted to advance our understanding. These findings offer insights into disease counseling, enhance clinical diagnosis, and may potentially pave the way for new therapeutic discoveries.

## Supporting information

S1 Fig Supplementary Figure 1 with captions. (DOCX)

S2 Fig Supplementary Figure 2 with captions. (DOCX)

S3 Fig Supplementary Figure 3 with captions. (DOCX)

S4 Fig Supplementary Figure 4 with captions. (DOCX)

S5 Fig Supplementary Figure 5 with captions. (DOCX)

S1 File Genotype Data Table. (XLSX)

## Abbreviations

(25(OH)D): 25-hydroxyvitamin D
AGRF: Australian Genome Research Facility
ANOVA: Analysis of variance
CI: Confidence intervals
CNS: Central nervous system
EBV: Epstein–Barr virus
HWE: Hardy-Weinberg Equilibrium
IRB: Institutional Review Board
MAF: Minor allele frequency
MS: Multiple sclerosis
OR: Odds ratios
RFLP: Restriction fragment length polymorphism
SNPs: Single Nucleotide Polymorphisms
VDR: Vitamin D receptor
