# Supplementary material for: VDR gene variants FokI and ApaI: Factors associated with susceptibility to multiple sclerosis
Source: PLoS One. 2025 Sep 17;20(9):e0332473. doi: 10.1371/journal.pone.0332473 (PMC12443253; doi:10.1371/journal.pone.0332473)
Supplement: S1 Fig — (DOCX) [file pone.0332473.s001.docx]

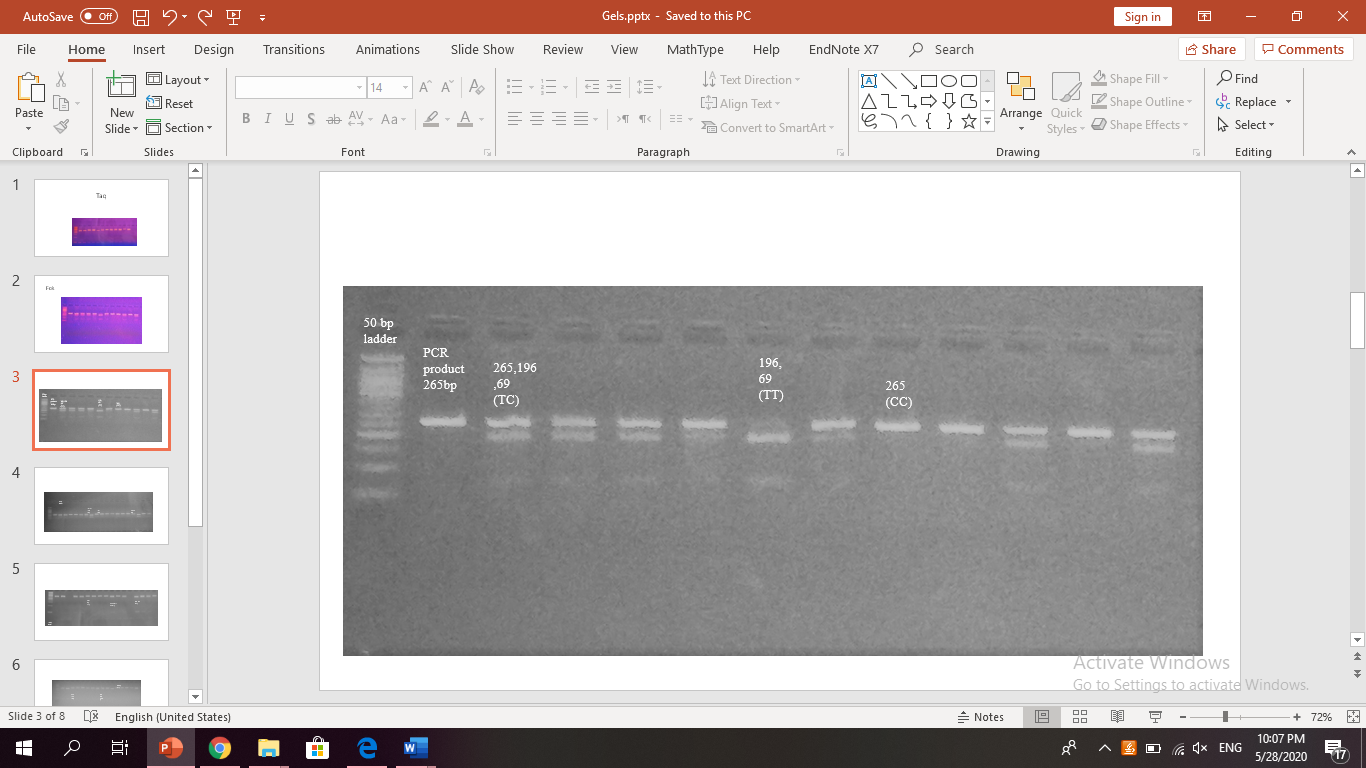


**Supplementary Figure 1:** Agarose gel electrophoresis showing different PCR-RFLP genotypes in the VDR gene according to the **FokI** SNP. The bands' size was determined by comparison to a 50 bp ladder. Lanes (1, 2, 3, 4 and 6) represent the heterozygous T/C genotype, with two bands at 196+ 69 bp for the T/ allele and one band at 265 for the C allele; lane 5 contains the homozygous T/T genotype, as indicated by two band at 196+ 69 bp; lane 7 genotype homozygous C/C one band at 265 bp.
