# Supplementary material for: VDR gene variants FokI and ApaI: Factors associated with susceptibility to multiple sclerosis
Source: PLoS One. 2025 Sep 17;20(9):e0332473. doi: 10.1371/journal.pone.0332473 (PMC12443253; doi:10.1371/journal.pone.0332473)
Supplement: S2 Fig — (DOCX) [file pone.0332473.s002.docx]

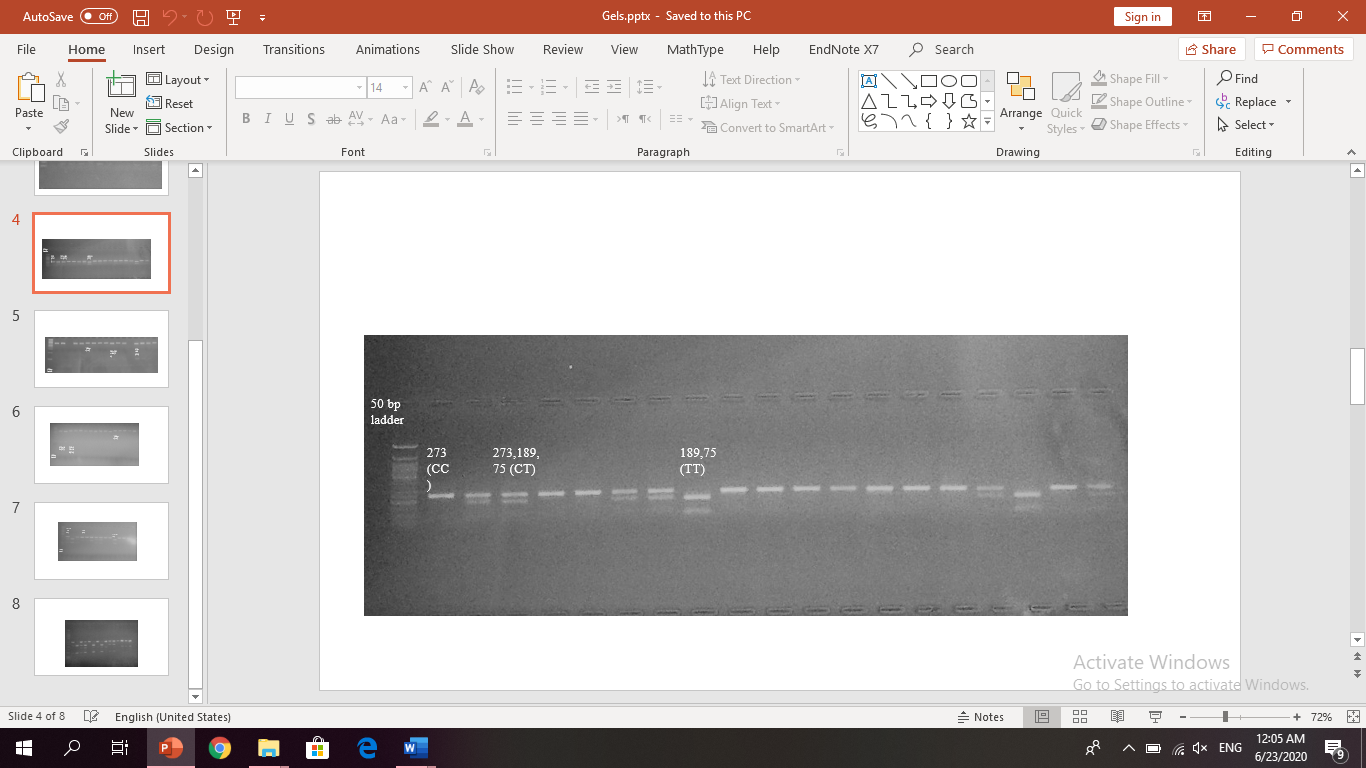


**Supplementary Figure 2:** Agarose gel electrophoresis showing different PCR-RFLP genotypes in the VDR gene according to the **BsmI** SNP. The bands' size was determined by comparison to a 50 bp ladder. Lanes (2, 3, 6 and 7) represent the heterozygous C/T genotype, with two bands at 189+ 75 bp for the T/ allele and one band at 273 for the C allele; lane 8 contains the homozygous T/T genotype, as indicated by two bands at 189+ 75 bp; lanes (1, 4, 5, 9 and 10) genotype homozygous C/C one band at 273bp.
