# Supplementary material for: VDR gene variants FokI and ApaI: Factors associated with susceptibility to multiple sclerosis
Source: PLoS One. 2025 Sep 17;20(9):e0332473. doi: 10.1371/journal.pone.0332473 (PMC12443253; doi:10.1371/journal.pone.0332473)
Supplement: S3 Fig — (DOCX) [file pone.0332473.s003.docx]

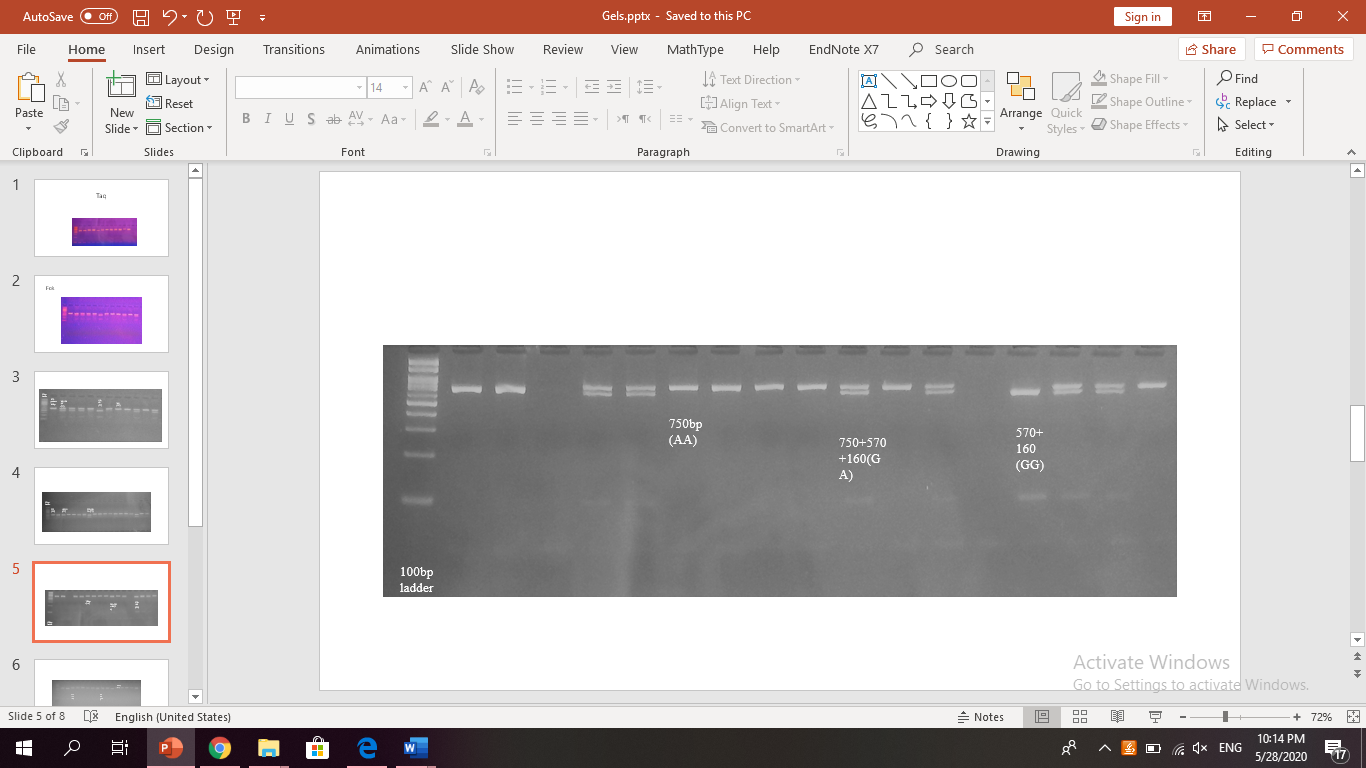


**Supplementary Figure 3:** Agarose gel electrophoresis showing different PCR-RFLP genotypes in the VDR gene according to the **TaqI** SNP. The bands' size was determined by comparison to a 100 bp ladder. Lanes (3, 4, 9, 11 and 13) represent the heterozygous G/A genotype, with two bands at 570+ 160 bp for the G/ allele and one band at 750 for the A allele; lane 12 contains the homozygous G/G genotype, as indicated by two bands at 570+ 160 bp; lanes (1, 2, 5, 6, 7, 8 and 10) genotype homozygous A/A one band at 750 bp.
