# Supplementary material for: VDR gene variants FokI and ApaI: Factors associated with susceptibility to multiple sclerosis
Source: PLoS One. 2025 Sep 17;20(9):e0332473. doi: 10.1371/journal.pone.0332473 (PMC12443253; doi:10.1371/journal.pone.0332473)
Supplement: S4 Fig — (DOCX) [file pone.0332473.s004.docx]

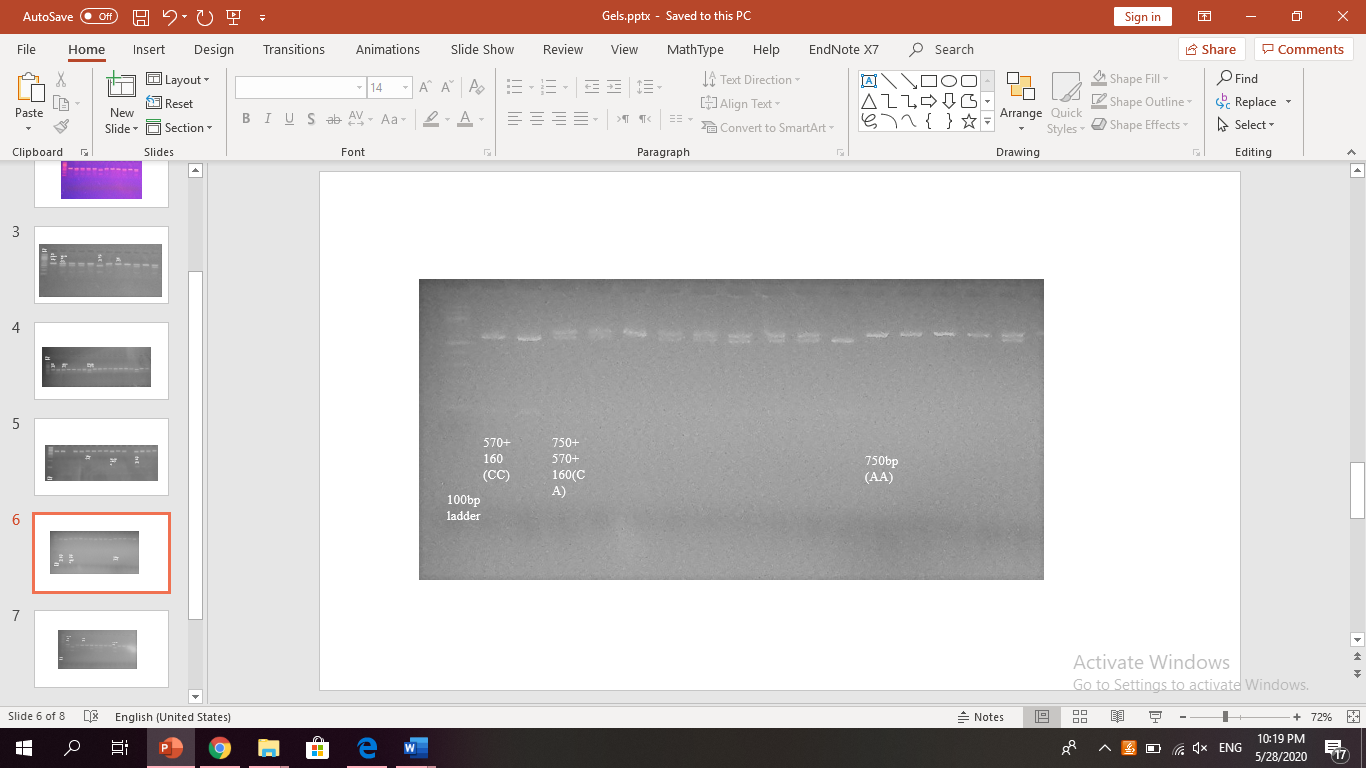


**Supplementary Figure 4:** Agarose gel electrophoresis showing different PCR-RFLP genotypes in the VDR gene according to the **ApaI** SNP (rs7975232). The bands' size was determined by comparison to a 100 bp ladder. Lanes (3, 4, 6, 7, 8, 9 and 10) represent the heterozygous C/A genotype, with two bands at 570+ 160 bp for the C/ allele and one band at 750 for the A allele; lanes (1, 2 and 11) contain the homozygous C/C genotype, as indicated by two bands at 570+ 160 bp; lane 12 genotype homozygous A/A one band at 750 bp.
