# Supplementary material for: VDR gene variants FokI and ApaI: Factors associated with susceptibility to multiple sclerosis
Source: PLoS One. 2025 Sep 17;20(9):e0332473. doi: 10.1371/journal.pone.0332473 (PMC12443253; doi:10.1371/journal.pone.0332473)
Supplement: S5 Fig — (DOCX) [file pone.0332473.s005.docx]

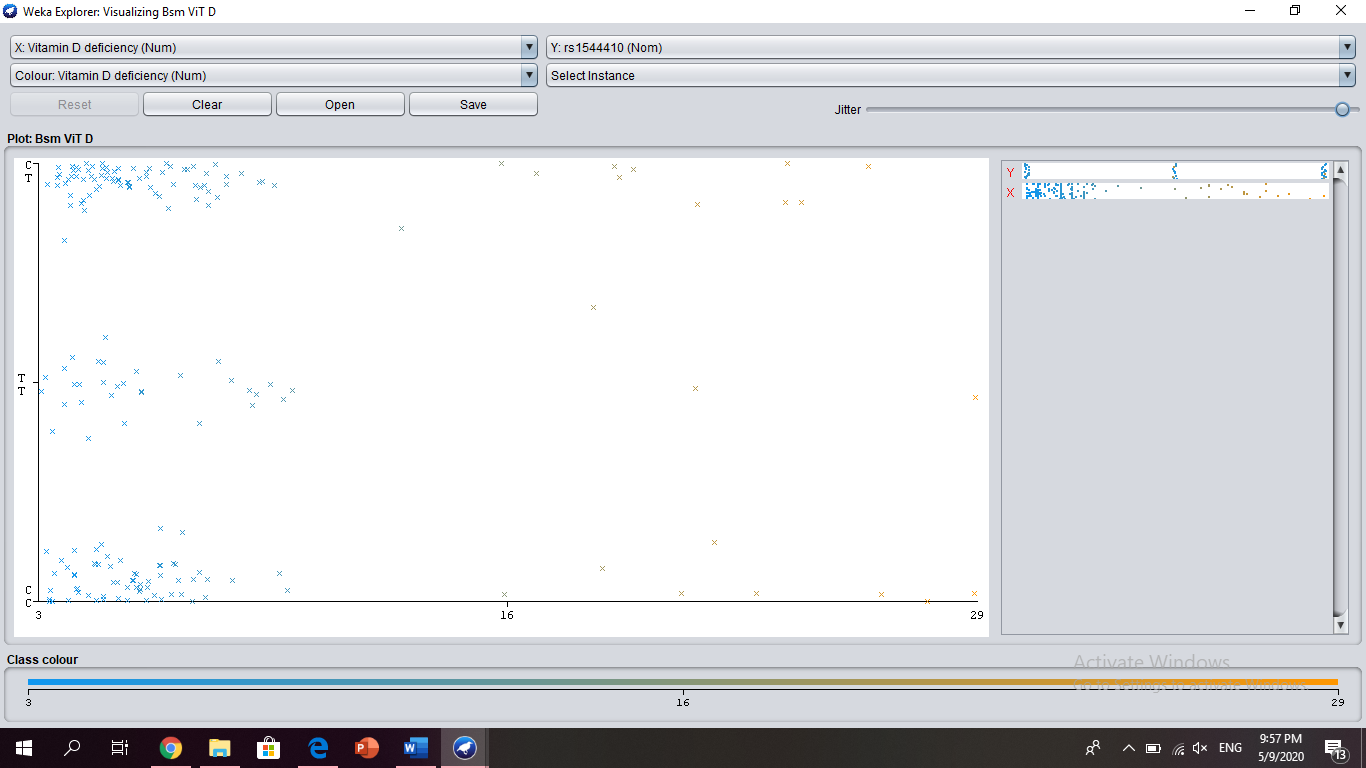


**A**


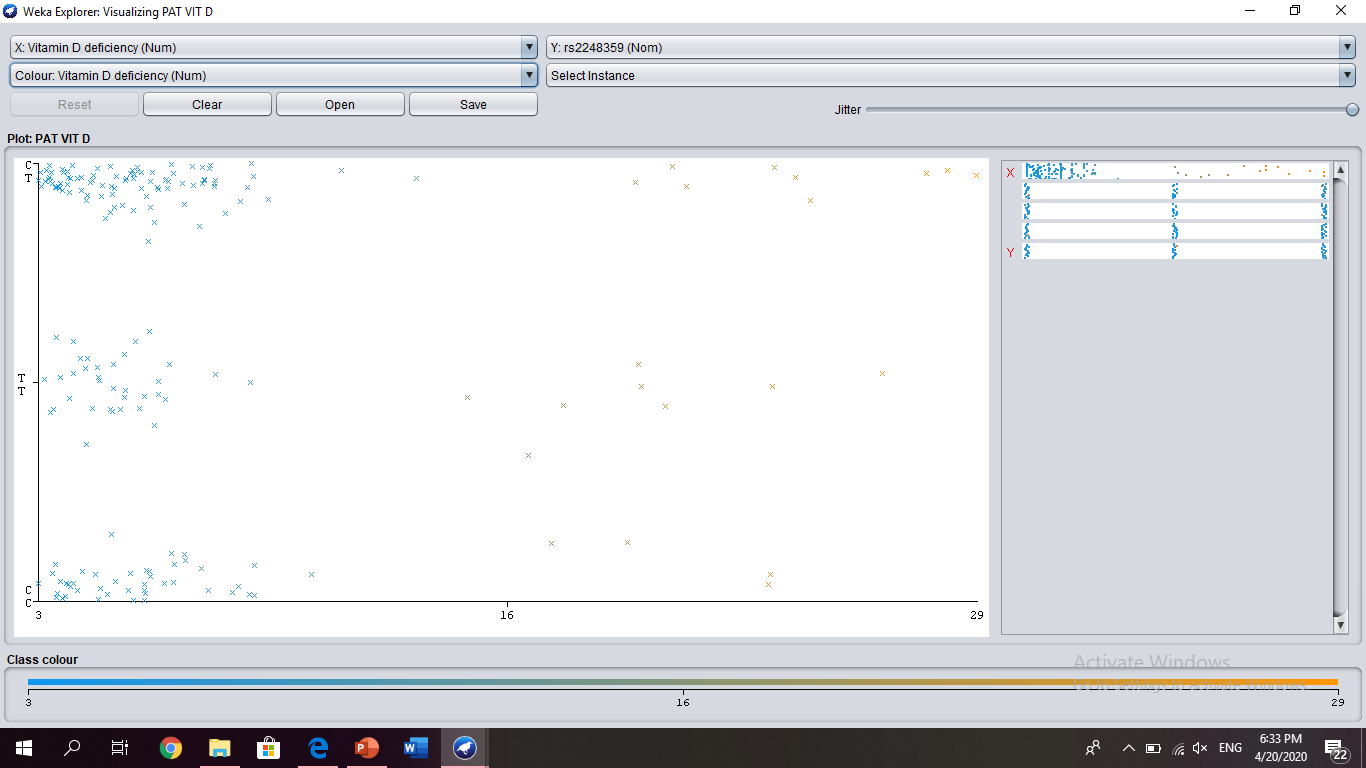


**B**


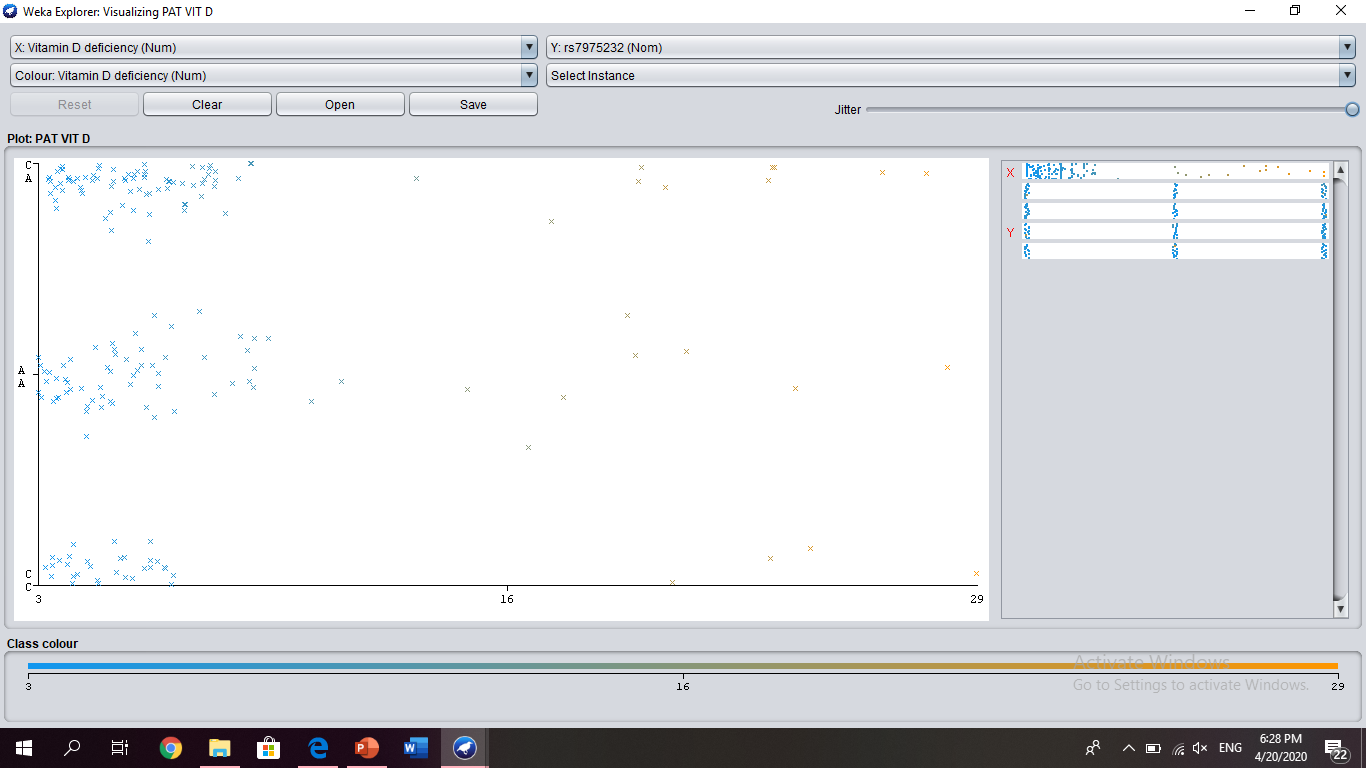


**C**


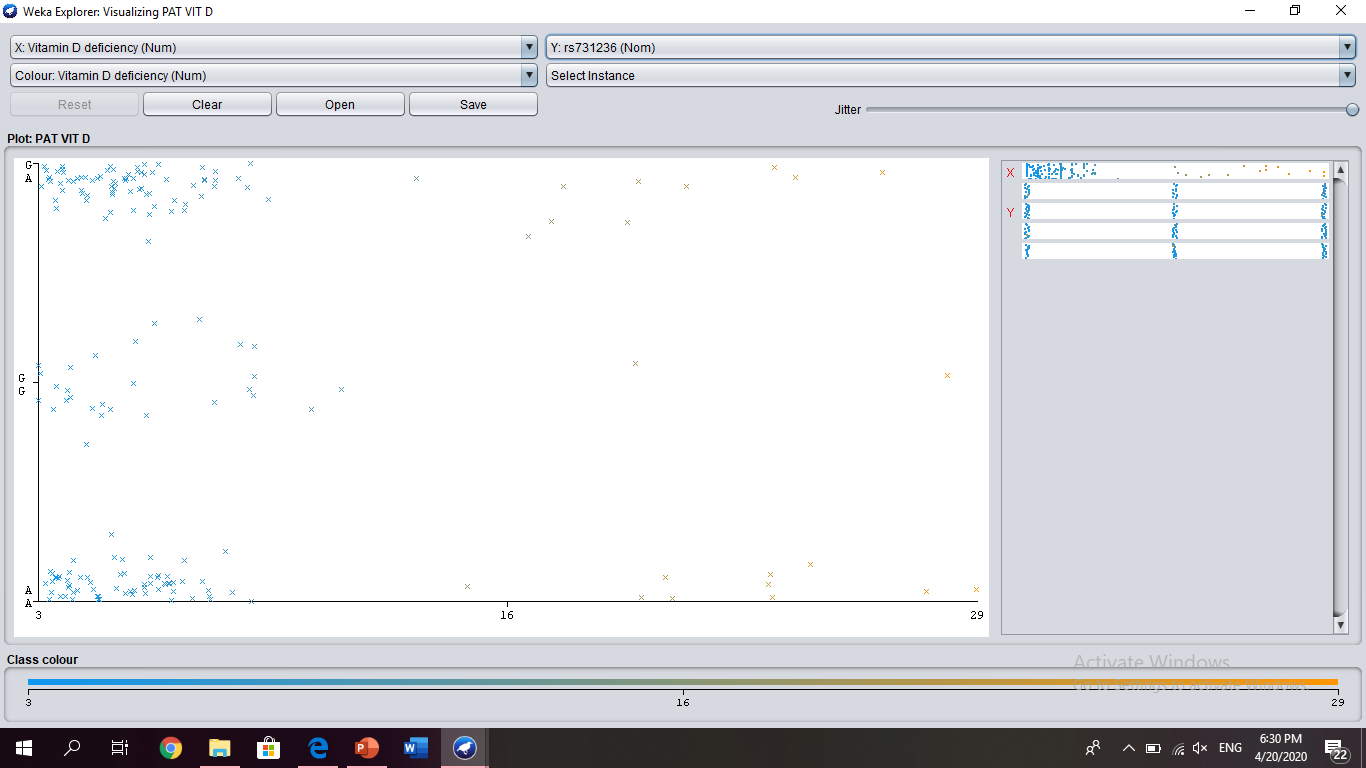


**D**

**Supplementary Figure 5**: The classification of Vitamin D levels with VDR SNP genotypes. (**A**: shows the relation between FokI genotypes and vitamin D levels of MS patients), (**B**: shows the relation between BsmI genotypes and vitamin D levels of MS patients), (**C**: shows the relation between ApaI genotypes and vitamin D levels of MS patients), and (**D**: shows the relation between TaqI genotypes and vitamin D levels of MS patients). X-axis: SNP genotypes, Y-axis: Vitamin D concentrations (3, 16 and 29 ng/ml).
